# 免疫组化染色评分对EGFR突变检测的影响

**DOI:** 10.3779/j.issn.1009-3419.2015.12.05

**Published:** 2015-12-20

**Authors:** 鑫 冯, 畅 刘, 殿胜 钟, 东波 徐, 超 宁, 杰 王

**Affiliations:** 1 300052 天津，天津医科大学总医院肿瘤内科 Department of Medical Oncology, Tianjin Medical University General Hospital, Tianjin 300052, China; 2 300070 天津，医科大学病理学教研室 Department of Pathology, Tianjin Medical University, Tianjin 300070, China

**Keywords:** 肺肿瘤, 免疫组化法, 表皮生长因子受体, 突变, Lung neoplasms, Immunohistochemistry, Epidermal growth factor receptor, Mutation

## Abstract

**背景与目的:**

恰当的免疫组化染色评分可确保突变检测结果的可靠性，目前大多数研究认为“四分法”是所有评分系统中最佳的方法。本研究旨在探讨不同四分法染色评分对检测结果的影响。

**方法:**

用三种不同的四分法免疫组化染色评分标准评价83例非小细胞肺癌标本表皮生长因子受体（epidermal growth factor receptor, EGFR）突变情况，并以液相芯片法作为金标准进行比较，计算每种方法的灵敏度、特异度、阳性预测值（positive predictive value, PPV）、阴性预测值（negative predictive value, NPV）、与金标准间一致度及三种标准间是否存在统计学差异。

**结果:**

三种标准在检测*EGFR*突变方面不存在统计学差异，每种标准检测结果的特异度均明显优于灵敏度。染色为“3+”的标本，PPV均可高达100%。

**结论:**

不同的四分法评分没有绝对的最佳标准，但无论用何种标准，免疫组化法（immunohistochemistry, IHC）检测*EGFR*突变的特异度均明显优于灵敏度。评分为“3+”的标本，可认为实际确实存在突变，直接接受*EGFR*酪氨酸激酶抑制剂（tyrosine kinase inhibitor, TKI）治疗。

## Abstract (en)

**Background and objective:**

Appropriate immunohistochemistry (IHC) scoring criteria can guarantee the reliability of mutation detection results. Most current studies suggest that the "four-grade criterion" may be the best among all scoring systems. The aim of this study is to discuss the influence of different four-grade immunohistochemistry scoring criteria on the test results.

**Methods:**

Three different four-grade immunohistochemistry scoring criteria were respectively used to evaluate the EGFR status of 83 cases of non-small cell of lung cancer (NSCLC) samples. The sensitivity, specificity, positive predictive value (PPV), negative predictive value (NPV), the agreement of each criterion compared with the gold standard, and whether statistical significance exists between each criterion were calculated.

**Results:**

No statistical difference was found among the three criteria in detecting *EGFR* mutations. All three criteria exhibit considerably better specificity than sensitivity. For samples with scores of "3+", PPV could reach up to 100%.

**Conclusion:**

No definite best criterion is among different four-grade scoring criteria. Regardless of the kind of criterion used, the specificity of IHC method in detecting *EGFR* mutations is markedly better than the sensitivity. For samples with scores of "3+", mutation status can be confirmed, and samples can receive EGFR-TKI therapy directly.

免疫组化法的染色评分是检测*EGFR*突变的最后一个步骤，但也是最重要的一步，因为恰当的评分系统可保证结果的可靠性。目前对于免疫组化染色尚无统一的评判标准，大部分方法都是以阳性细胞百分比与染色强度相结合的方式评判^[1-4]^。应用较多的是Yu等^[1]^提出的四分法：其将结果分为0-3+四个等级，以肿瘤细胞的胞膜和/或胞浆染色为基准，0为肿瘤细胞无染色或 < 10%的肿瘤细胞浅染; 1+为 > 10%的肿瘤细胞浅染; 2+为肿瘤细胞中度染色; 3+为肿瘤细胞强染。1+-3+为阳性，0为阴性。此后，Simonetti等^[5]^、Hofman等^[6]^也应用同样方法判读结果。此外，还有将染色强度与阳性细胞百分比相乘的方法，以结果大于某一界定数值为阳性，小于该数值为阴性，如Colorado大学的免疫组化H评分法^[2, 7]^和Kozu等^[8]^所使用的方法。目前大多数实验研究认为基于肿瘤细胞胞膜和/或胞浆的染色强度与染色区域的百分比设定的评分标准，并按程度分为0-3+四个等级，是所有评分系统中最佳的方法^[4]^。

2013年，Jiang等^[4]^沿袭了四分法的基本框架，但对每一级别的染色强度和阳性区域百分比进行了更为详尽的描述，相比Yu等^[1]^对2+（中度染色）及3+（强染）级别仅设定笼统强度指标的方法更为严谨。本文通过比较三种四分法评分标准及各自与金标准间的一致度，来探讨四分法中每一级别或阴性阳性的不同界定给检测结果带来的影响。

## 1 材料与方法

### 1.1 研究对象

收集天津医科大学总医院2010年12月-2012年10月收治的非小细胞肺癌患者手术切除或组织活检标本97例，上述标本均已接受EGFR抗E746_A750del突变单克隆兔抗体（6B6）和抗L858R突变单克隆兔抗体（43B2）的免疫组化染色，经筛选有43例可查到对应标本的液相芯片法检测结果; 另新收集了我院2012年2月-2013年9月经液相芯片法检测验证的存在EGFR 19或21外显子突变（各包含20例）的手术组织标本40例（与之前标本无重叠），经天津医科大学总医院病理科核对、切片、行特异性抗体的免疫组化染色制成病理片子。最终获得既有EGFR液相芯片法检测结果，亦有免疫组化染色病理片子的标本总共83例，标本取材前患者均未经EGFR酪氨酸激酶抑制剂（tyrosine kinase inhibitor, TKI）治疗，包含腺癌68例、大细胞肺癌6例、鳞癌9例。

### 1.2 免疫组织化学染色步骤

切片常规二甲苯脱蜡，经各级浓度乙醇水化。取一定量pH6.0柠檬酸盐缓冲液（北京中杉金桥生物技术有限公司），加入微波盒中，医用微波炉中火加热切片3 min×2次进行抗原修复，凉至室温40 min。每张切片加1滴3% H_2_O_2_，室温下孵育10 min。加1滴稀释倍数为1:100的第一抗体（抗E746_A750del突变单克隆兔抗体（6B6）和抗L858R突变单克隆兔抗体（43B2）; 美国Cell Signaling Technology公司），4 ℃冰箱过夜。而后加辣根酶标记的第二抗体（北京中杉金桥生物技术有限公司），37 ℃温箱30 min-40 min。每张切片加1滴新鲜配制的DAB液显色20 min，用淡苏木素复染细胞核30 s，各级浓度乙醇快速脱水，二甲苯透明，中性树脂封片。晾干后观察^[9, 10]^。

### 1.3 染色评判标准

对83例标本的免疫组化病理片子行3种判读方法，染色评分均以肿瘤细胞染色强度及肿瘤细胞胞膜和/或胞浆阳性区域百分比为基准，将染色结果分为0-3+四个等级。A法为综合若干文献^[1, 8, 11]^，并经我院病理医师确认后所定标准：0：无染色或 < 10%肿瘤细胞任意强度染色; 1+：10%-50%肿瘤细胞浅染或中度染色; 2+：10%-50%肿瘤细胞强染; 3+： > 50%肿瘤细胞任意强度染色。以0为阴性，1+、2+、3+为阳性。B法为参考文献Jiang等^[4]^所用标准：0：无染色; 1+：肿瘤细胞浅黄染不伴明显颗粒或不超过10%的肿瘤细胞黄染伴明显颗粒; 2+：超过10%的肿瘤细胞黄染伴明显颗粒或不超过10%的肿瘤细胞棕染伴明显颗粒; 3+：超过10%的肿瘤细胞棕染伴明显颗粒。对于B法，分别以1+和2+为阳性分界点，将其分为B1（0为阴性，1+-3+为阳性）和B2（0、1+为阴性，2+、3+为阳性）两种方法。评分由天津医科大学总医院病理科三位老师各自单独进行，结果经汇总，对有差异的评分经复核商讨后得到最终一致结果。

### 1.4 统计学分析

统计学分析应用统计学软件SPSS V21.0（SPSS Inc.）进行。以液相芯片法为金标准，将其检测结果为19外显子缺失突变和21外显子点突变的作为实际阳性病例，将野生型作为实际阴性病例，计算A法、B1法和B2法三种免疫组化判读标准检测上两种突变各自的灵敏度、特异度、阳性预测值（positive predictive value, PPV）、阴性预测值（negative predictive value, NPV）。应用Cohen κ值计算三种免疫组化评判标准各自与金标准间的一致度：κ值介于0.81-1认为一致性最强，介于0.61-0.80为高度一致，介于0.41-0.60为中度一致，介于0.21-0.40为一致性尚好，介于0.00-0.20为轻度一致。对于每一个κ值，计算其95%可信区间（confidence interval, CI），若每种方法κ的95%CI上限及下限无重叠，则认为各种方法间的差异有统计学意义（*P* < 0.05）。

## 2 结果

### 2.1 三种不同免疫组化评分标准评价*EGFR*突变结果分析

83例有液相芯片法检测结果的标本中：57例（57/83, 68.7%）存在*EGFR*基因突变，其中28例（28/83, 33.7%）存在19外显子缺失突变，30例（30/83, 36.1%）存在21外显子点突变，各包括1例（1/83, 1.2%）既存在19外显子缺失突变、亦存在L858R点突变的标本; 另外26例（26/83, 31.3%）为野生型*EGFR*。

### 2.2 根据*EGFR*突变类型进行统计

在57例突变标本中，IHC法检测阳性为：A法26例，B1法30例，B2法17例; 在26例野生型标本中，IHC法检测阴性为：A法23例，B1法21例，B2法24例。特异性抗体免疫组化法检测上两种突变在A法中灵敏度为45.6%，特异度为88.5%，PPV为89.7%，NPV为42.6%。B1法中灵敏度为52.6%，特异度为80.8%，PPV为85.7%，NPV为43.8%;B2法中灵敏度为29.8%，特异度为92.3%，PPV为89.5%，NPV为37.5%。三种方法各自与金标准间的一致度Cohen’s Kappa（95%CI）在A法中为0.264（0.134, 0.410）; B1法为0.272（0.116, 0.464）; B2法中为0.158（0.025, 0.301）。B1法较A法、B2法与金标准间的一致度更高，但差异无统计学意义（*P* > 0.05）（表 1）。

**1:** 三种IHC评分方法检测*EGFR*突变的相关数据比较 Comparison of three different criteria in detecting *EGFR* mutations by IHC

Criteria	Sensitivity	Specificity	PPV	NPV	Cohen’s Kappa 95%CI
A	45.6%	88.5%	89.7%	42.6%	0.264 (0.134, 0.410)
B1	52.6%	80.8%	85.7%	43.8%	0.272 (0.116, 0.464)
B2	29.8%	92.3%	89.5%	37.5%	0.158 (0.025, 0.301)
PPV: positive predictive value; NPV: negative predictive value; EGFR: epidermal growth factor receptor; IHC: immunohistochemistry.					

在28例实际存在19外显子缺失突变的标本中，E746_A750del特异性抗体的IHC法检测阳性为：A法14例，B1法16例，B2法10例; 在余55例实际不存在19外显子缺失突变的标本中，IHC法检测阴性为：A法52例，B1法50例，B2法53例。免疫组化法在A法中灵敏度为50.0%，特异度为94.5%，PPV为82.4%，NPV为78.8%;B1法中灵敏度为57.1%，特异度为90.9%，PPV为76.2%，NPV为80.6%;B2法中灵敏度为35.7%，特异度为96.4%，PPV为83.3%，NPV为74.6%。三种方法各自与金标准间的一致度Cohen’s Kappa（95%CI）在A法中为0.493（0.282, 0.661）; B1法为0.512（0.321, 0.692）; B2法中为0.373（0.114, 0.580）。B1法较A法、B2法与金标准间的一致度更高，但结果无统计学意义（*P* > 0.05）（表 2）。

**2:** 三种IHC评分方法检测19外显子缺失突变的相关数据比较 Comparison of three different criteria in detecting EGFR E19del by IHC

Criteria	Sensitivity	Specificity	PPV	NPV	Cohen’s Kappa 95%CI
A	50.0%	94.5%	82.4%	78.8%	0.493 (0.282, 0.661)
B1	57.1%	90.9%	76.2%	80.6%	0.512 (0.321, 0.692)
B2	35.7%	96.4%	83.3%	74.6%	0.373 (0.114, 0.580)

在30例实际存在21外显子点突变的标本中，L858R特异性抗体的IHC法检测阳性为：A法12例，B1法14例，B2法7例; 在余53例实际不存在21外显子点突变的标本中，IHC法检测阴性为：A法53例，B1法53例，B2法53例。免疫组化法在A法中灵敏度为40.0%，特异度为100.0%，PPV为100.0%，NPV为74.6%;B1法中灵敏度为46.7%，特异度为100.0%，PPV为100.0%，NPV为76.8%;B2法中灵敏度为23.3%，特异度为100.0%，PPV为100.0%，NPV为69.7%。三种方法各自与金标准间的一致度Cohen’s Kappa（95%CI）在A法中为0.460（0.265, 0.657）; B1法中为0.528（0.331, 0.719）; B2法中为0.280（0.071, 0.461）。B1法较A法、B2法与金标准间的一致度更高，但差异无统计学意义（*P* > 0.05）（表 3）。

**3:** 三种IHC评分方法检测21外显子点突变的相关数据比较 Comparison of three different criteria in detecting *EGFR* E21 mutation by IHC

Criteria	Sensitivity	Specificity	PPV	NPV	Cohen’s Kappa 95%CI
A	40.0%	100.0%	100.0%	74.6%	0.460 (0.265, 0.657)
B1	46.7%	100.0%	100.0%	76.8%	0.528 (0.331, 0.719)
B2	23.3%	100.0%	100.0%	69.7%	0.280 (0.071, 0.461)

### 2.3 根据免疫组化染色程度逐层分析

如表 4所示，当以A法为评判标准时：54例评分为“0”的标本中，31例实际存在19或/和21外显子突变，概率为57.4%（31/54）; 13例评分为“1+”的标本中，10例实际存在突变，概率为76.9%（10/13）; 5例评分为“2+”的标本中，实际全部存在突变，概率为100%（5/5）; 11例评分为“3+”的标本中，实际全部存在突变，概率为100%（11/11）。

**4:** 以A法为评判标准的IHC染色结果逐层分析 Analysis of each staining degree based on criterion A

IHC-A			Liquid chip technology (gold standard)			
Scores	*n*		E19del mutation	E21 mutation	Percentage of mutation	Wt (%)
0	54		14*	18*	57.4% (31/54)	23 (42.6%)
1+	13		5	5	76.9% (10/13)	3 (23.1%)
2+	5		1	4	100% (5/5)	0 (0%)
3+	11		8	3	100% (11/11)	0 (0%)
^*^Including one case that has both E19del and E21 mutations.						

同样地，如表 5所示，当以B法为评判标准时：48例评分为0的标本中，27例实际存在19或/和21外显子突变，概率为56.3%（27/48）; 16例评分为“1+”的标本中，13例实际存在突变，概率为81.3%（13/16）; 9例评分为“2+”的标本中，7例实际存在突变，概率为77.8%（7/9）; 10例评分为“3+”的标本中，实际全部存在突变，概率为100%（10/10）。

**5:** 以B法为评判标准的IHC染色结果逐层分析 Analysis of each staining degree based on criterion B

IHC-B			Liquid chip technology (gold standard)			
Scores	*n*		E19del mutation	E21 mutation	Percentage of mutation	Wt (%)
0	48		12*	16*	56.3% (27/48)	21 (43.8%)
1+	16		6	7	81.3% (13/16)	3 (18.8%)
2+	9		7	0	77.8% (7/9)	2 (22.2%)
3+	10		3	7	100%（10/10）	0（0%）
^*^Including one case that has both E19del and E21 mutations.						

## 3 讨论

本文以液相芯片法检测结果作为金标准，比较了三种四分法染色评判标准（A、B1、B2），结果显示，B1法优于A法、B2法，但差异无统计学意义（*P* > 0.05）。另外，无论用何种评判标准，免疫组化法检测*EGFR*突变的特异度明显高于对应灵敏度。这一结果也与近些年来大宗文献报道情况相符^[6, 11-13]^。

对于*EGFR* 19或21外显子突变的检测，B1法有优于B2法的趋势，此结果与蒋贵阳文章中的结果相反。究其原因，考虑与抗原修复液pH值不同有关：由于抗原在不同pH值环境中，等电点会发生改变，抗原抗体表面电荷的改变影响二者结合，造成在不同pH值的抗原修复液中染色强度不同^[14, 15]^。本实验所用抗原修复液为pH 6.0的柠檬酸钠溶液，Jiang等^[4]^所用为pH 9.0的EDTA溶液。熊焰等^[11]^研究了不同抗原修复液对免疫组化染色结果的影响，提示pH6.0的柠檬酸钠抗原修复液使肿瘤细胞染色相对较浅，可能致阳性染色不易识别出，此时使用灵敏度更高的“1+”作为阳性分界有助于阳性标本的识别，故本实验中B1法较B2法与金标准间一致度更高; 而pH9.0的EDTA抗原修复液可使肿瘤细胞着色较强，非特异性着色亦较强^[11]^，此时提高阳性标准，以“2+”为阳性分界的方法可减少检测的假阳性率，增加其与金标准间的一致度。熊焰^[11]^提出，使用pH8.0的EDTA抗原修复液可使组织切片显色最佳，即特异性染色强而背景色浅。

根据免疫组化染色程度逐层分析，结果显示，“0分”层，应用A法与B法评价野生型的概率相差甚微（42.6% *vs* 43.8%），但有将近60%实际突变的标本未能检测出，所以应借助灵敏度更高的分子水平检测手段进一步评价; “1+”层中，两种方法预测*EGFR*突变概率（76.9% *vs* 81.3%）抑或野生型概率（23.1% *vs* 18.8%）差异亦不大。对于“2+”层，应用A法预测*EGFR*突变率可高达100%，而B法为77.8%，这是因为A法的“2+”评分（10%-50%肿瘤细胞强染）对阳性要求更高，已相当于B法的“3+”评分（超过10%的肿瘤细胞强染），因此“2+”评分层中，A法比B法的特异度更高。另外，B法的“2+”评分中检出突变的概率（77.8%）低于“1+”（81.3%），考虑可能是少数突变只能表现出弱阳性，按特异度要求更高的“2+”标准反而不能检测出，而降低了突变检出率; 还可能与“2+”中样本量较少有关。在“3+”层中，无论对于A法还是B法，预测*EGFR*突变率均可高达100.0%，此结果亦与众多文献报道相符^[4, 5]^。由此可得出，虽然免疫组化染色方法会漏诊相当一部分实际突变标本，但提高染色评分标准可确保突变检测的准确性，尤其对于评分为“3+”的标本，结果可靠，实际确实存在突变，直接接受EGFR-TKI治疗。

不同的四分法评分没有绝对的最佳标准，同一标准因受不同染色试剂等因素的影响，结果可出现差异，但无论用何种染色标准进行评判，免疫组化法检测*EGFR*突变的特异度均明显优于灵敏度。对于新评分法的选用，与传统经典方法比较并校正可确保新方法的可靠性。提高染色评分标准可确保突变检测的准确性，尤其对于评分为3+的标本，结果可靠，直接接受EGFR-TKI治疗。
